# Acute Psychosis as the First Manifestation of Neuro-Behçet's Disease

**DOI:** 10.7759/cureus.116559

**Published:** 2026-09-19

**Authors:** Mohamed Hamid, Ahmed Bourazza

**Affiliations:** 1 Neurology, Military Hospital Moulay Ismail of Meknes, Sidi Mohamed Ben Abdellah University, Fez, MAR; 2 Neurology, Mohammed V Military Hospital, Rabat, MAR

**Keywords:** acute psychosis, corticosteroids, mesencephalon, neuro-behçet’s disease, psychiatric disorders

## Abstract

Behçet’s disease is a chronic, recurring systemic inflammatory vasculitis of unknown etiology. If the central nervous system is involved, the condition is referred to as neuro-Behçet’s disease. In addition to classical neurological involvement, patients with Behçet’s disease may present with complex neurobehavioural and mental disorders, which are termed neuro-psycho-Behçet’s disease. We describe a rare case of a 28-year-old man presenting with acute psychosis as the initial presentation of neuro-Behçet’s disease.

## Introduction

Behçet's disease (BD) is a persistent, recurrent, systemic inflammatory vasculitis with an indeterminate cause. BD is traditionally defined by the presence of recurring oral and genital ulcers, cutaneous lesions, and uveitis. It may also present as a multisystem condition impacting important systems [1]. When the central nervous system (CNS) is affected, the disorder is referred to as neuro-Behçet’s disease (NBD). Neurological symptoms manifest in 3%-30% of individuals with BD, generally appearing many years after the first systemic symptoms [2].

NBD is grouped into two primary types: parenchymal involvement, typically manifesting as subacute meningoencephalitis or subacute brainstem syndromes, and non-parenchymal involvement, usually causing cerebral venous sinus thrombosis [3]. In addition to classic neurological impairments, individuals with BD may exhibit a multifaceted neurobehavioral and mental condition known as "neuro-psycho-Behçet’s disease" (NPBD) [4]. We report an uncommon case of a man with acute psychosis as the primary presentation of NBD.

## Case presentation

A 28-year-old male, previously in excellent health, was brought to our emergency department exhibiting severe agitation. He possessed neither a personal nor familial history of mental health issues or psychosocial elements that may have precipitated a psychotic episode. Based on his family's report, he developed psychomotor agitation and hostile behavior two hours before admission. For four days, he had been complaining of a severe headache, loss of appetite, and a gradual loss of communication.

The patient’s vital signs were normal on admission. On physical examination, we noticed inflammatory lesions in the oral and genital areas but no neck stiffness. Neurological examination revealed minor dysarthria and mild left-sided hemiparesis. Deep tendon reflexes were increased with a positive left-sided Babinski sign. The ophthalmologic examination revealed posterior uveitis without papilledema. A mental status examination indicated poor attention, disorganized thinking, echolalia, and unrelated language. The assessment of the other extra-neurological system was normal.

Laboratory tests were unremarkable (hemogram, renal and hepatic tests, C-reactive protein test, serum electrolytes, and thyroid hormone). A toxicological study concluded no drugs or toxic chemicals. The pathergy test was positive for BD. Cranial magnetic resonance imaging (MRI) showed hyperintense lesions in the mesencephalon, putamen-pallidum complex, right mesial temporal lobe, and right internal capsule (Figures 1, 2, 3).

**Figure 1:**
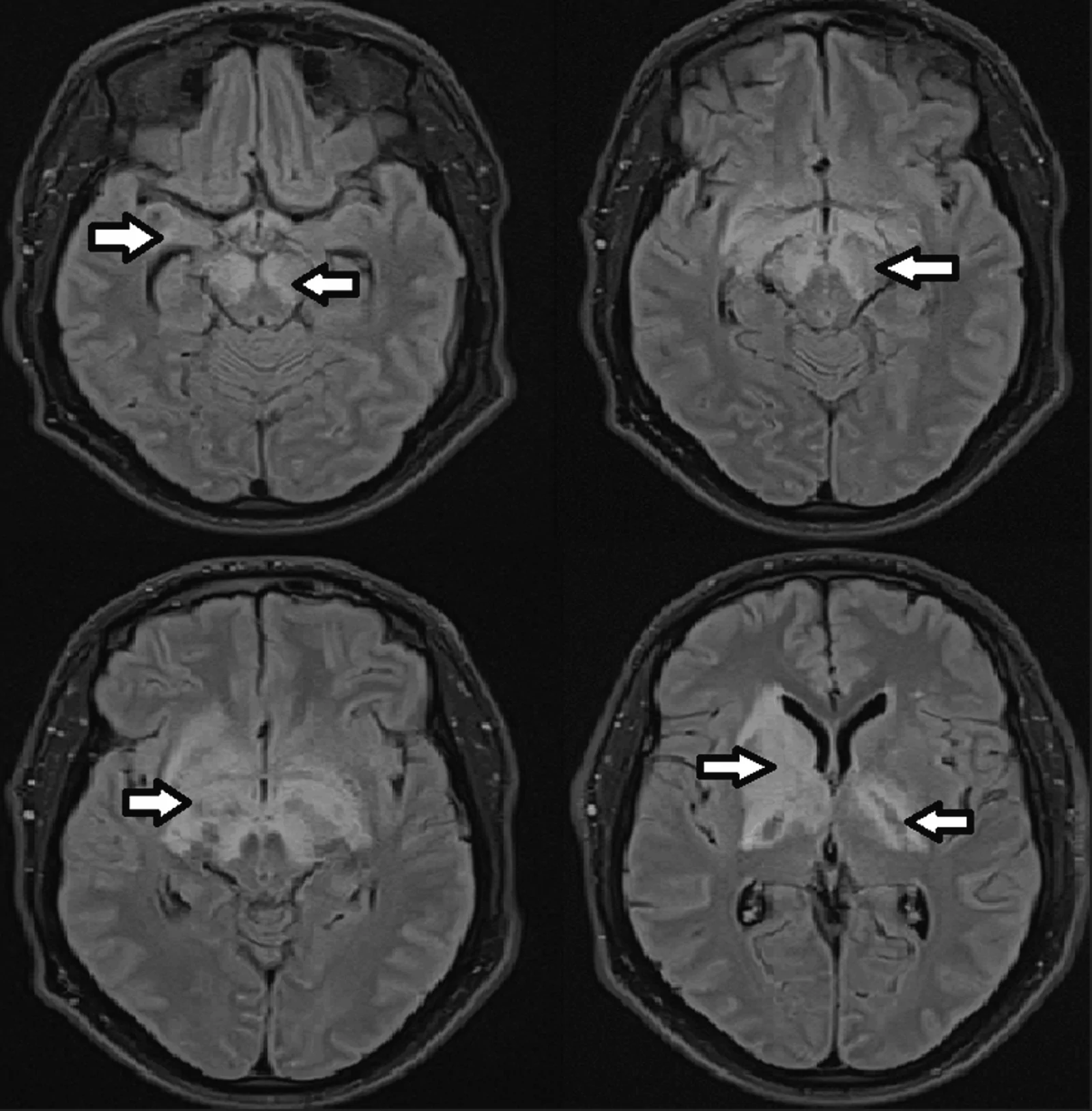
Cranial MRI on axial fluid-attenuated inversion recovery (FLAIR) sequences showing high signal intensity in cerebral peduncles, right medial temporal and both lenticular nuclei (arrows).

**Figure 2:**
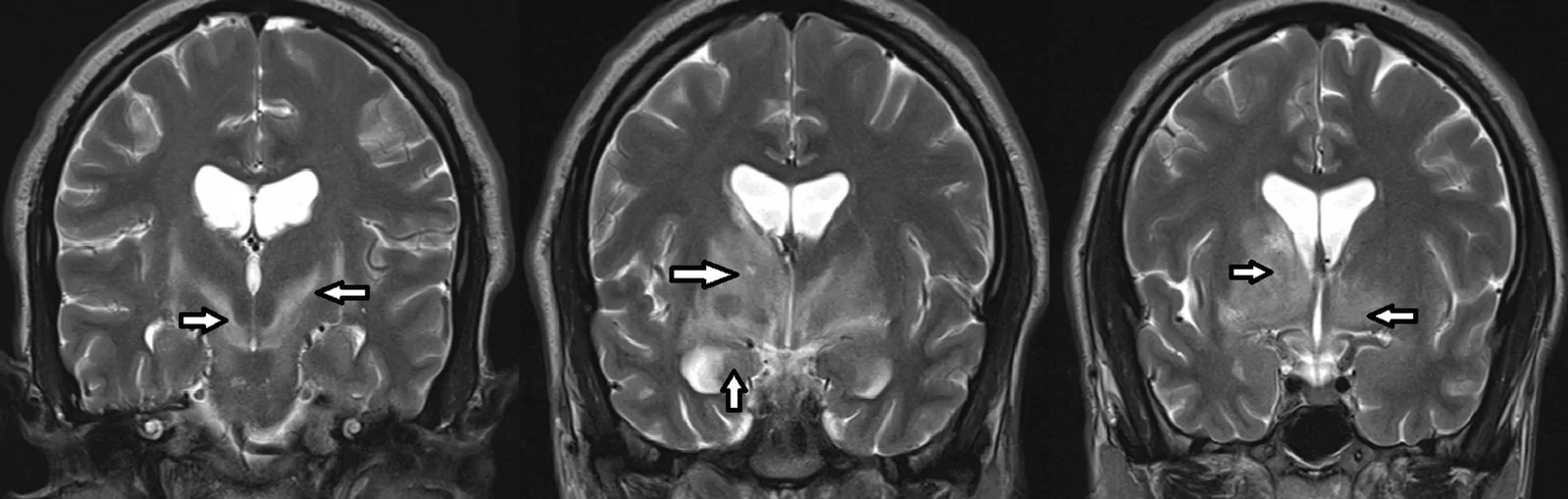
Cranial MRI on coronal T2-weighted sequence reveals bilateral asymmetric hyperintensity in the midbrain and the putamen-pallidum-capsular complex (arrows).

**Figure 3:**
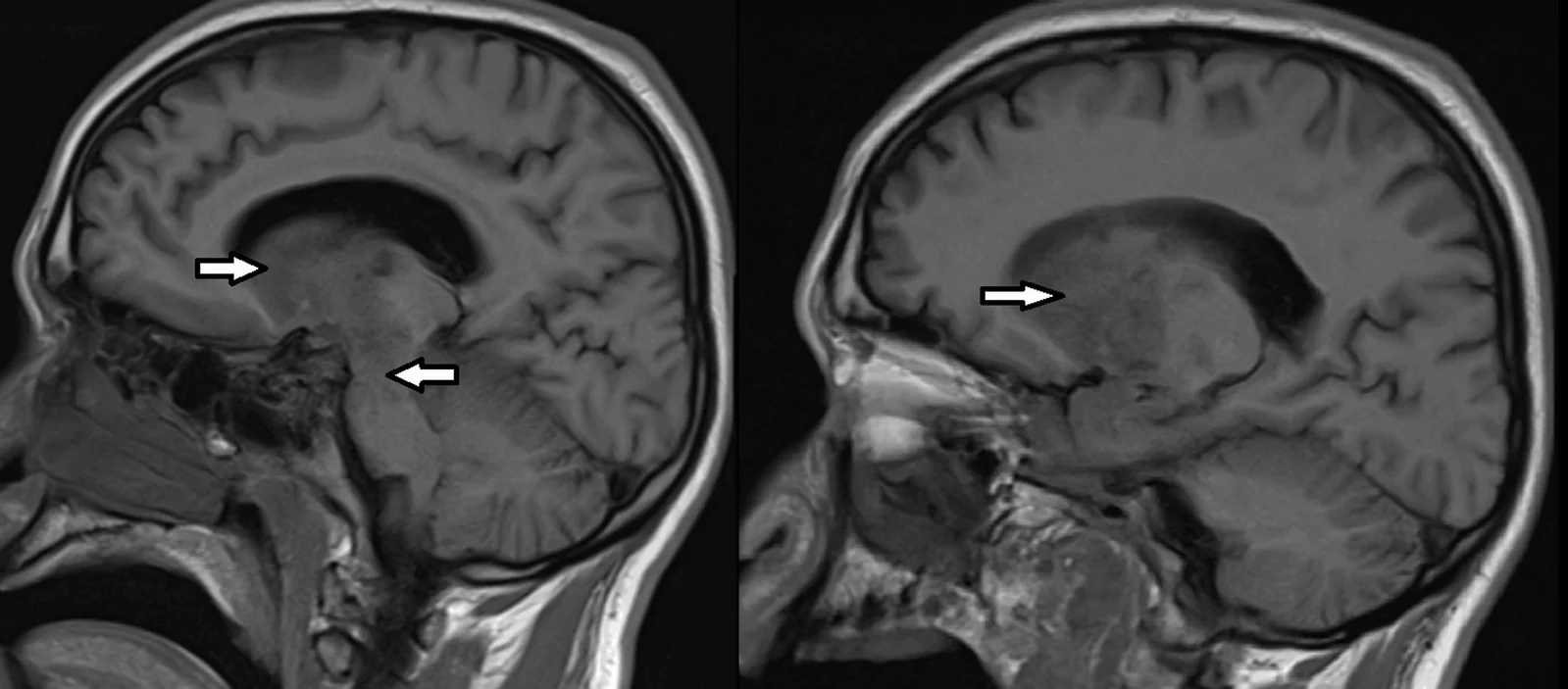
Cranial MRI on sagittal T1-weighted sequence shows low signal intensity in the mesencephalon-diencephalon region (arrows).

The MRI revealed enhancement on contrast-enhanced T1-weighted images in the right internal capsule and right putamen-pallidum complex (Figure 4). The venous MRI angiography was normal.

**Figure 4:**
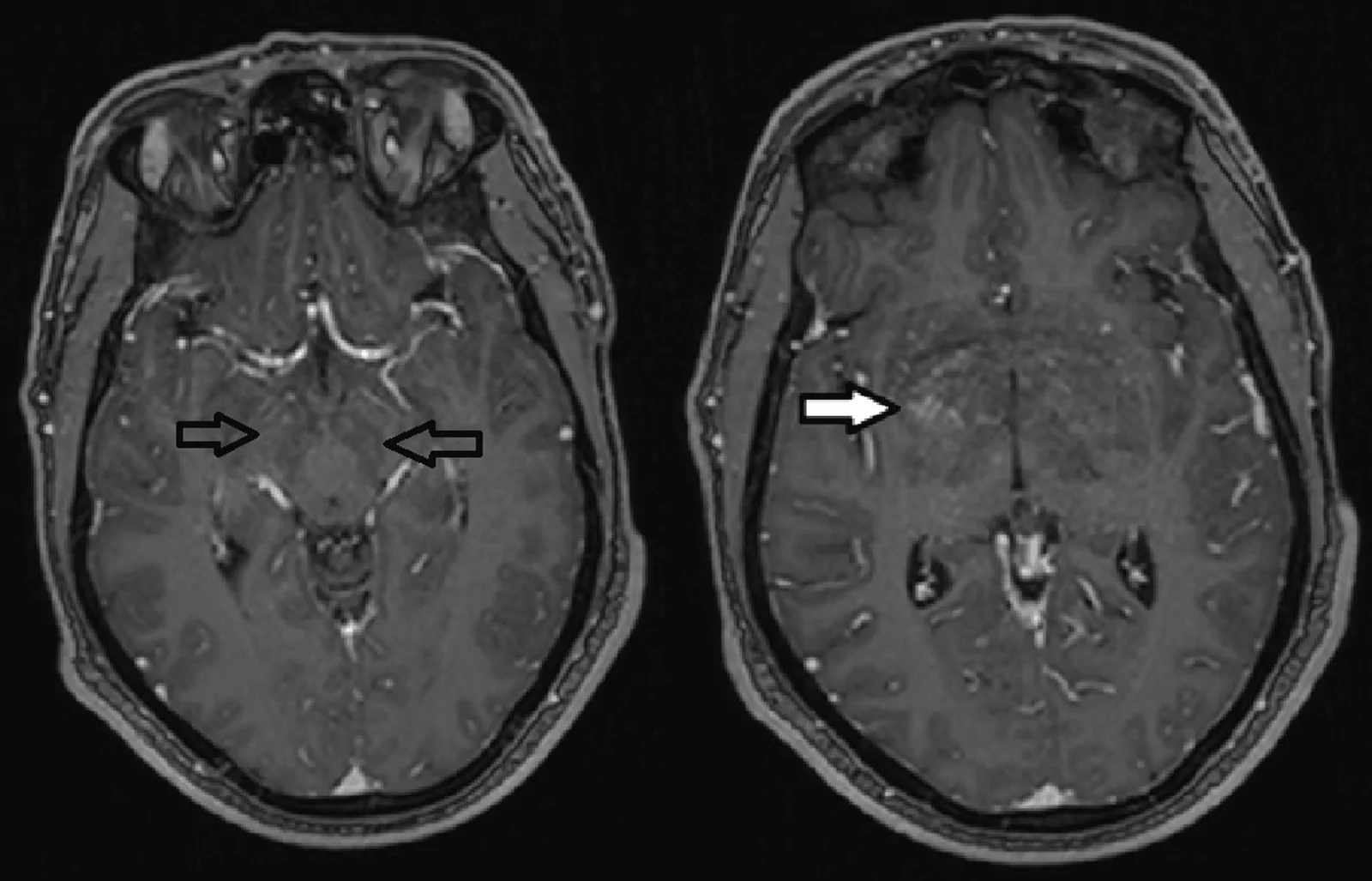
Cranial MRI on contrast-enhanced T1-weighted images demonstrates patchy areas of enhancement in the right capsular-putamen-pallidum complex (white arrow), without midbrain enhancement (black arrows).

The electroencephalogram (EEG) results were unremarkable. The cerebrospinal fluid (CSF) contained 28 leukocytes/μL with 85% of lymphocytic cells and a total protein concentration of 75.2 mg/dL. Results of infectious screenings in CSF for syphilis, herpes simplex virus (HSV), Epstein-Barr virus (EBV), Borrelia, cytomegalovirus (CMV), varicella-zoster virus (VZV), and toxoplasmosis were negative. Autoimmune disease tests were negative.

Based on the findings from the clinical assessment, which include oral and genital ulcers, ocular lesions, a positive pathergy test, neurological deficits, psychotic episode, and mental state examination disorders correlated with MRI and CSF abnormalities, and after ruling out alternative diagnoses, our patient was diagnosed with NPBD.

Intravenous methylprednisolone boluses (1 g/day) were prescribed to our patient for a duration of five days, followed by 1 g/day weekly for four weeks, along with intravenous cyclophosphamide (1 g/month). Colchicine was administered with an oral steroid regimen starting at 80 mg/day and progressively reduced to a minimum of 5 mg/day. Risperidone and benzodiazepines were used for managing psychomotor agitation and psychological disorders. Physiotherapy was employed to correct the motor weakness. The patient's psychiatric disorders improved after two weeks of hospitalization. The patient had minor residual left hemiparesis with no systemic problems or recurrence after three months of follow-up.

## Discussion

NPBD is a clinical construct proposed to characterize individuals with bipolar disorder or other psychiatric manifestations. It is categorized as a descriptive framework rather than a rigorous diagnostic classification, although it reflects a clinically and pathophysiologically complex concept. It is clinically and immunopathogenically similar to other autoimmune and neuroinflammatory conditions with neuropsychiatric involvement, such as multiple sclerosis, systemic lupus erythematosus, and anti-NMDA receptor encephalitis [4].

The clinical recognition of psychiatric symptoms in BD has evolved over several decades. Over five decades ago, Epstein et al. presented the initial description of BD with psychiatric aspects in 1970 [5]. Despite its historical identification, NPBD has received little rigorous research, which has resulted in considerable deficiencies in our comprehension of its varied clinical presentations and etiopathology.

The etiology of NPBD is increasingly understood using a biphasic framework that connects vascular neuroinflammation with neurochemical impairment. The inflammatory-neurotransmitter phase corresponds to parenchymal neuroinflammation induced by systemic immune dysregulation in BD. Increased levels of pro-inflammatory cytokines, such as interleukin-6 (IL-6), tumour necrosis factor-alpha (TNF-α), and T-helper 17 (Th17)-related mediators, have been identified to impair the integrity of the blood-brain barrier [6]. The structural phase is marked by chronic microvascular inflammation in the venules, leading to macroscopic parenchymal damage and gliosis [7].

The anatomic localization of these parenchymal lesions shows a significant correlation with the psychological effects. The primary etiology of psychotic symptoms could be explained by neuroinflammatory alterations in the basal ganglia, capsulostriatal regions, and limbic structures. Behavioral disorders and obsessive-compulsive symptoms are related to changes in the frontostriatal circuit [1,8].

The manifestation of NPBD is generally multiphasic, with systemic BD signs followed by neurologic abnormalities and cognitive symptoms. Neurological signs may include hypoesthesia, hemiparesis, dysmetria, spasticity, and headaches; psychiatric signs may range from manic episodes to severe depressive states and psychotic crises [9].

Structural neuroimaging (MRI) usually shows T2-hyperintense parenchymal lesions in the brainstem, internal capsule, and basal ganglia [10]. EEG abnormalities may be expressed as temporal lobe focal activity. CSF analysis often reveals inflammatory pleocytosis and elevated protein concentrations [1].

A diagnostic approach for NPBD has been proposed to guide clinical assessment: (1) Validate the diagnosis of BD using the International Criteria for Behçet's Disease (ICBD) [11]; (2) The presence of a neuropsychiatric condition characterized by central nervous system involvement (neurological signs or paraclinical evidence from CSF, MRI, or EEG) alongside at least one notable mental symptom (psychosis, mood disorders, or changes in behavior/personality); (3) Temporal association between neuropsychiatric symptoms arising concurrently with or after the diagnosis of BD; (4) Elimination of alternative causes, including fundamental psychiatric conditions, current infections, demyelinating disorders (such as multiple sclerosis), and substance abuse [1].

Some case studies report a link between psychiatric disorders and NBD. Aydin et al. reported a case of NBD accompanied by hypomania [12]. Nkam and Cotterau discovered that a patient with NBD underwent a psychotic episode in the absence of parenchymal brain injury [13]. Deniz et al. documented a case involving a young woman with acute psychosis associated with NBD [10].

Successful management of NPBD requires a multidisciplinary collaborative approach combining autoimmune and psychiatric therapies. High-dose corticosteroids are used to treat acute neuroinflammatory episodes followed by a progressive oral discontinuation protocol [14]. Immunosuppressive drugs (azathioprine or cyclophosphamide) and biologic agents such as anti-CD20 therapy (rituximab) or TNF-alpha antagonists (infliximab) [13,15] are needed for persistent control. Psychiatric treatment is adapted to the phenotypic presentation of symptoms. Olanzapine, quetiapine, and risperidone are examples of atypical antipsychotics used to treat psychotic episodes. Mood stabilisers (sodium valproate, carbamazepine, or lithium) are used for treating bipolar disorders [16,17].

The long-term outcome of NPBD is rather variable and is generally viewed as poor because of the progressive nature of parenchymal CNS damage. Some patients obtain partial or complete clinical remission using combination regimens, but many experience persistent cognitive abnormalities, learning issues, or irreversible motor deficits. The worst cases can be lethal, for example, multi-system or extensive brainstem involvement [1].

## Conclusions

NBD is a complicated multi-system neurobehavioral condition with CNS inflammation that may overlap with a spectrum of mental symptoms. The case presented here shows the clinical and etiopathogenic complexity of BD with neuropsychiatric manifestations. A major diagnostic issue is recognizing psychiatric manifestations as sentinel or early indicators of NBD, as symptoms may precede or be concurrent with neuroclinical signs and lead to serious misdiagnoses.
